# Iranian nursing students' experiences of workplace violence: a qualitative study

**DOI:** 10.5249/jivr.v15i1.1655

**Published:** 2023-01

**Authors:** Ebrahim Aliafsari Mamaghani, Azad Rahmani, Hadi Hassankhani, Vahid Zamanzadeh, Sue Dean, Alireza Irajpour, Arman Azadi

**Affiliations:** ^*a*^ Department of Nursing, Maragheh University of Medical Sciences, Maragheh, Iran.; ^*b*^ Department of Medical Surgical Nursing, School of Nursing and Midwifery, Tabriz University of Medical Sciences, Ta-briz, Iran.; ^*c*^ Faculty of Health, Southern Cross University, Queensland, Australia.; ^*d*^ Department of Critical Care Nursing, School of Nursing and Midwifery, Isfahan University of Medical Sciences, Isfahan, Iran.; ^*e*^ Social Determinants of Health Research Center, Isfahan University of Medical Sciences, Isfahan, Iran.; ^*f*^ Nursing Department, School of Nursing and Midwifery, Ilam University of Medical Sciences, Ilam, Iran.

**Keywords:** Workplace violence, Nursing students, Vertical violence, Horizontal violence, Clinical education

## Abstract

**Background:**

Workplace violence against nursing students is a common phenomenon. This study aimed to investigate Iranian nursing students’ experiences of workplace violence, including their reaction to violence and the consequences and the effects of such violence on the students.

**Methods:**

The study participants were undergraduate nursing students. The data were collected using semi-structured, in-depth face-to-face interviews. Data analysis was carried out with qualitative approach using conventional content analysis.

**Results:**

Four categories were extracted from the analysis of the interview transcriptions: vertical violence, horizontal violence, reaction to violence and consequences of violence. Nurses were the major imposers of violence against students and psychological and verbal violations were the most of used forms of violence. The students reacted to violence in the forms of counteracting, reporting, disregarding and considering as commonplace.

**Conclusions:**

Workplace violence is a common phenomenon experienced by nursing students in this study, which causes devastating individual, educational, and professional impacts. Action plans including providing safe environment and appropriate support from nurses and educators should be developed in clinical settings to intervene and to prevent workplace violence.

## Introduction

Because of the nature of their occupation, nurses are the main victims of workplace violence.^1-3^ The high prevalence of workplace violence against nurses and nursing students has been an international concern^4,5^ and advanced countries have been facing this challenge as well.^4,6,7^ While there have been many studies on clinical violence towards staff nurses than nursing students; related studies showed differences in the types, contributing factors and results of clinical violence between the two groups.^6,8,9^ Nursing students experience workplace violence vertically from nurses, clinical educators, and physicians as well as from patients and their companions or horizontally from their classmates.^4,10^ However, in different societies, cultural and social grounds, as well as nursing education systems are factors influencing type, the extent of violence and even individuals’ definition of violence.^11^

Although some studies have been conducted on the consequences of workplace violence against nurses,^12,13^ these outcomes have been less researched among nursing students. The results of these studies have also revealed that experiencing workplace violence can lead to psychological complications such as anxiety, depression, decreased self-esteem, irritability, feelings of helplessness and fear^6,14,15^ and damages to the students’ physical health.^14^ Furthermore, the experience of workplace violence can also result in the poor quality of care provided by nursing students, reduced professional commitment^14,16^ the degradation of students' professional values and identity^6^ and quitting the nursing profession by students in some cases.^14^

Some studies conducted in Iran^17,18^ and other Middle Eastern countries, including Turkey,^19-21^ have reported that nursing students experience more verbal violence^11,18,21^ and that the physical violence experienced by the students is mostly imposed by the patients and their companions.^18,21,22^ Nevertheless, workplace violence in the clinical setting is underreported. Nurses and nursing students are reluctant to report violence from patients because it may be considered as a normal part of job. They are also very hesitant to report violence from classmates or seniors. In addition, little information is available on some aspects of the workplace violence against nursing students, i.e. the students’ reactions to experiencing violence and the consequences of the violations. On the other hand, such knowledge is of paramount importance to develop any guidelines to reduce the incidence of workplace violence against students and support programs for the victims of workplace violence.

The violence against Iranian nurses has been well published, but few, have focused on how nursing students perceive such violence. The aim of this study was to examine the experiences of Iranian nursing students of workplace violence, including their reactions to experiencing violence and the consequences of the violations for these students.

## Methods

***The study design and context***

To describe the studied phenomenon, the conventional content analysis was used. This study was conducted at Tabriz Nursing Faculty affiliated to Tabriz University of medical sciences. This university is located in the East Azarbaijan Province, the northwest of Iran. At the time of sampling, about 800 undergraduate students were studying at this college.

***Participants***

The participants were 20 undergraduate nursing students. The inclusion criteria were: (i) being nursing student; (ii) passing the fourth semester or more; and (iii) willingness to enroll in the study. The first three students were selected using convenient sampling method. These three students had many experiences regarding the studied phenomenon and had the tendency and ability to transfer these experiences. Other subjects were selected using purposive sampling approach based on the analysis of previous interviews. The samples were selected in such a way that, while observing the principles of purposive sampling, they were highly diverse in terms of gender, years of education, educational status, and cultural background.

***Data collection***

The data were collected using semi-structured, in-depth face-to-face interviews during August to January in 2017-2018. The interviews were conducted after informed consent was received in settings where the participants were willing to attend. Interviews with 12 participants were done in private rooms at clinical settings and other interviews were held in a private room at nursing school.

To initiate each interview, some warm-up questions were first asked to reduce stress and create trust among the participants. In the following, the main questions were raised: What kinds of workplace violence have you experiences? Who performed such behaviors to you? How often do you experience such situations in clinical settings? What was your reaction in those situations? What were the consequences of such situations? Considering the participants’ responses to the aforementioned questions, the next questions were asked with the purpose of probing as follows: How? When? Who? What was the result? Please further explain. Describe it with an example.

All interviews were performed and analyzed by the first author (A. A.) who was trained in conducting qualitative interviews in Persian language. The interviews were recorded for further accurate implementation. Some statements expressed by the participants were translated semantically into English to be included in this paper. The first 20 interviews lasted for an average of 65 minutes (40-110 minutes) and the other nine complementary interviews lasted for an average of 22 minutes (18-35 minutes). As the topic of the interview was the concern of most of participants, the time of the interviews was lasted longer than usual. The follow-up interviews were done with some of the participants to confirm the findings of the initial interviews or to eliminate some ambiguities.

***Data analysis***

To understand the main points of the interviews, each interview was listened to several times before the recorded file was transcribed. Subsequently, the interviews were imported as separate words into the MAXQDA software version 10. 

The interview transcriptions were simultaneously analyzed during the data collection phase. To ensure that codes were grounded in the participants’ real experiences, data were subjected to content analysis using the following steps proposed by Graneheim and Lundman.^23^

1. The interviews were transcribed verbatim and read through several times to gain a sense of the whole.

2. The text was divided into units of meaning that were then further condensed. 

3. The condensed meaning units were then summarized and labeled with codes.

4. The codes were organized into subcategories and categories, based on comparisons regarding their similarities and differences.

5. Finally, themes were developed as the expression of the latent content of the text.

This process continued until the main and secondary categories were formed. Eventually, four main categories were obtained.

***Data trustworthiness***

To validate the data, four criteria (namely credibility, transferability, confirmability and dependability) proposed by Lincoln and Guba (1985) were concerned.^24^ Credibility was reached through allocating a long time to data collection and confirming the findings with the help of the three participants. Dependability of the data was accepted by comparing the evaluation results of three independent researchers regarding the codes presented for two interviews. Confirmability of the findings was accomplished through accurate recording of all the documents relevant to the compilation and analysis of the findings. Transferability was made possible through searching for contradictory items, sampling with the highest diversity and confirming the findings by three nursing students who were not sampled.

## Results

Twenty Iranian nursing students aged between 20 and 22 years (mean (SD): 21.50 (0.81)) were interviewed (Table 1 ). 

**Table 1:** Characteristics of participants and interviews.

Variables	Mean±SD	Min-max	N (%)
**Age**	21.50±0.81	20-22	
**Gender**			
Female			8 (40.0)
Male			13 (60.0)
**Interview duration **(minute)	65.00±16.14	40-110	
**Trimester**			
4			4 (20.0)
5			3 (15.0)
6			5 (25.0)
7			4 (20.0)
8			4 (20.0)
**Interview place**			
Nursing school			12 (60.0)
hospital			8 (40.0)

The analysis of the interviews led to the emergence of four categories: (1) Vertical violence; (2) Horizontal violence; (3) Reaction to violence; (4) Consequences of violence (Table 2).

**Table 2:** Category and sub category of nursing students' experiences of workplace violence.

Categories	Main properties
**Vertical violence**	Vertical violence imposed by health personnel
	Vertical violence imposed by patients and their companions
**Horizontal violence**	Violence by one of the clinical group members
	senior students violence
**Reaction to violence**	Counteracting
	Reporting
	Disregarding
	Considering as commonplace
**Consequences of violence**	Physical and psychological damages
	Educational outcomes
	Professional outcomes

***Vertical violence***

Vertical violence included two sub-categories of violence imposed by health personnel and violence imposed by patients and their companions.

***Vertical violence imposed by health personnel***

Due to the frequent need to communicate with nurses, the students experienced the most violence on the part of clinical nurses. Lack of time, high workload, and negative attitude towards nursing students were among the most important causes of violence posed by the nurses. The prevalence of violence experienced from the nurses was higher among students who were more interested in communication with the nurses as well as female students.

*"Nurses consider us as an extra load. They always tell us that they didn’t have time. They do not even have time to say hello."*

The most common types of violence imposed by the nurses were verbal and psychological, including ignoring students, calling students inappropriately, shouting at students, disregarding nursing students, fussing over what students do, disrespecting, humiliating or mocking students. In a few cases, the violence by the nurses was of a physical nature, such as pushing or jostling the students.

In few cases, the clinical educators had also imposed psychological and rarely verbal violence against students. These include calling students violently, blaming students for their mistakes, and humiliating them. The violence was mostly imposed by low-experienced educators having low clinical expertise.

 Although students did not report violence on the part of other health personnel including physicians, they reported several cases of disrespect, insults, and discrimination on the part of the service personnel, especially the police personnel.

*"I wanted to take my car into the hospital. I had an argument with the guard. He showed me disrespect."*

***Vertical violence imposed by patients and their companions***

Patients and their companions were the major sources of violence against nursing students and the most frequent types of violence imposed by them against students was verbal, including calling students inappropriately or calling the student violently. In two cases, students also experienced physical violence, i.e. pushing.

*"I was in the ward talking to a nurse. A patient yelled at me and said “Don’t you hear? I have pain in my body."*

The students had noticed that the most important causes of violence by the patients and their companions were lack of treatment facilities, poor quality of care, resistance of patients to some therapeutic measures, imposed pain and suffering on patients caused by students’ lack of expertise, and not allowing the companions to meet their patients. The experience of this kind of violence was more frequent in female and junior students.

*"The patient did not let me to get blood. He shouted at me to stop doing that since he thought his blood was gone."*

**Horizontal violence**

***Violence by one of the clinical group members***

Students' training in clinical groups of 5 to 7 persons provides the grounds for violence imposed by classmates and other nursing students. In most cases, the violence was of a psychological and verbal type and involved calling inappropriately, yelling, humiliating one another in the presence of the instructor, medical staff or patients. In some cases, the violence was of a cultural type and students from different cultures or students living in large cities ridiculed students from small towns or villages. 

*"Not a big deal now! In the first semesters, I couldn’t say where I come from since my classmates used to pull my leg."*

***Senior students’ violence***

 Although a great amount of the violence was imposed by the classmates, the senior students were sometimes the cause of the violence. Additionally, most of the violence was imposed by the students of the same gender, even though; it was also observed among the male students as well. Eventually, when the students in the clinical groups were of the same gender, the incidence of such violence increased.

*"When all boys are in a group, they annoy others but girls are dealing with each other more easily."*

**Reaction to Violence**

The students reacted to violence in the forms of counteracting, reporting, disregarding and considering as commonplace.

***Counteracting***

Some students counteracted the imposed violence. Although this method was employed more often for the patients and their companions, it was also used to deal with nurses and other health care personnel. Aggression, insults, verbal conflict, disrespect, and shouting were among the most frequent coping strategies used by the students. This was especially true about senior and male students.

*“We, students, also responded harshly to the head nurse told him to repeat his words respectfully not to insult."*

Counteracting makes the educators blame the students because the educators do not intend their relations with nurses to be damaged. Further, the nurses also label these students as inflexible and refuse to cooperate with them. Hence, observing these problems makes other students unwilling to use this method.

*"Our instructor also supported the nurse [causing violence] because he did not want to be in odds with the staff."*

***Reporting***

Reporting violence to clinical educators and, in some cases, to the head nurses was the students’ another reaction to workplace violence. Some students, usually female and junior students, used this method to respond to the violence due to their lack of self-confidence. Another group of students were those who first counteracted in response to the violence; however, they later decided to report the cases of violence because of inefficiency of the former method or the educators’ insistence. 

*"I inform my instructor of any violence because he's in charge of us and I trust him."*

After reporting the cases of violence, the educators usually try to resolve the problem by replacing the patient in cases where the source of violence is patients or their companions. The cases of severe violence are reported to the hospital's police forces. Reporting was not effective in cases where the source of violence was the nurses themselves, causing the students to be more blamed.

*"I once had an argument with a patient in the emergency ward and the instructor told me not to take care of that patient anymore."*

***Disregarding***

Some students gradually learn that counteracting or reporting are not useful strategies to deal with violence. On the other hand, due to the lack of other strategies, students try not to react in spite of experiencing high internal tension against the imposed violence. Indeed, students are convinced that nurses have the absolute power and they are only guests in the clinical setting. The most important causes of disregarding violence are low self-esteem, fear of being fired from the wards, being known as a troublemaker and getting low clinical scores.

*"I'm afraid of the nurses. They have clinical wards. They rampage when we make mistakes."*

Considering the patients, the students also gradually found that disregarding their violence is safer with respect to the patient's health condition. However, the students are less likely to use this technique when dealing with the patients’ companions. In these cases, they mostly use counteracting or reporting approaches.

*"If you fight with the patient, he maybe gets worse and a trouble appears so it's better to say nothing and keep calm.”*

***Considering as commonplace***

After a while, some students who first reported or counteracted to violence or disregarded it assume that workplace violence is commonplace in their profession and they must accept it. Witnessing this behavior among some clinical educators and even nurses also strengthen such an attitude in students.

*"There are conflicts in all wards and nothing can be done. It must be accepted that this is commonplace in nursing."*

 This attitude is especially common amongst the senior students who have had many experiences of workplace violence not being pursued. The violence imposed by the nurses, not by patients, their companions, and other health care personnel, were mostly considered as commonplace.

*"I'm to be graduated this year. I have experienced such behaviors a lot. It's okay and you should admit it. He is a nurse and we are students."*

**Consequences of Violence**

The consequences of violence against students included physical and psychological damages, educational outcomes, and professional outcomes.

***Physical and psychological damages***

One of the consequences of violence against students is physical and psychological damage. Due to the low prevalence of physical violence, workplace violence rarely led to direct physical harm to students and the observed cases of physical damage were the result of psychological stress associated with violence. The most important physical damages were insomnia, nightmares, and headaches, loss of appetite, nausea, and stomach burn. 

*"Once I had a fight with a sick person, I was so stressed out that I had nausea and vomiting by night."*

The damages were more intense in female students and those who were psychologically weaker. Additionally, the incidence of these complications was higher among the students who did not use counteracting or disregarding methods to face with violence. On the contrary, these complications were less severe among the students who were poorly educated and had lower levels of educational motivation.

*"I do not have the desire to learn such healthcare matters. I do not care about how others treat."*

***Educational outcomes***

Experience of workplace violence causes negative educational outcomes such as frequent absence from work, delayed arrival, and frequent requests for rapid departure from the clinical environment by the students. The long-term impact of experiencing violence is to reduce the student's motivation for nursing care as well as to decrease their confidence in performing their tasks. 

*"Because of a simple mistake in registering a file, the nurse treated me so badly that I am afraid of working with patients."*

If nurses were the source of the violence, such experiences would make students be afraid of interaction with nurses and thus lose a large number of educational opportunities. Male students and students who experience violence imposed by medical staff are more likely to experience negative educational outcomes.

*"A nurse shouted at me because of a negligible mistake at the surgery department and I did speak to no other nurse until the end of that clinical course."*

***Professional outcomes***

Experience of workplace violence can also lead to negative professional outcomes, one of which is the reduced quality of nursing care provided by students due to their reduced motivation to care for patients. Interestingly, some students reported that the experience of violence on the part of nurses make them no to ask their questions from nurses even if they were in doubt.

*"That day after the conflict, my mind was so obsessed that I could not even adjust the serum droplets let alone diagnosing the patients' problems."*

The experience of violence would deteriorate the students’ professional attitude and this, especially in the first semesters, partially changes students’ attitudes towards nursing profession to the extent that they decide not to pursue the nursing profession. In addition, the experience of violence and different treatment to medical students reduces the professional relationship between nursing students and medical students and ignites the envy of nursing students towards these students. Any insult or disrespect to the student also made them doubt about their profession and future career. The student preferred to remain unemployed to work in the department where he was insulted or offended.

*"I prefer unemployment to being employed in the digestion ward since the staff behaved the nurses too offensively."*

## Discussion

This study is one of the few studies examining the issue of workplace violence against nursing students, with an emphasis on students' reactions to the violence and the consequences of violence against nursing students in Iran and other Middle Eastern countries.

The findings of this study indicated that the nurses were the major imposers of violence against nursing students. Studies in Australia^25^ and Turkey^21^ considered patients to be the most important source of violence against nursing students. This finding evidently contradicts with the findings of the present study. The negative attitude of Iranian nurses’ towards nursing students and not considering students as a member of the medical team^26-28^ can lead to a high incidence of violence by nurses against nursing students. Interestingly, the patients' companions were assumed as another major source of violence against nursing students in this study. This point was less mentioned in previous studies. In Iran, there is always a companion with most patients, who is often a member of the patient's family and stays with the patient during the entire length of hospitalization.

The findings showed that the experienced violence was mainly verbal and psychological. However, workplace violence rarely led to direct physical harm to students and the observed cases of physical damage were the result of psychological stress associated with violence. The incidence of these complications was higher among the students who did not use counteracting or disregarding methods to face with violence. Regarding the type of violence experienced, previous studies have also shown that verbal and psychological violence is the most common type of violence experienced by nursing students,^4,11,29^ which is consistent with the findings of this study.

The findings of this study, as the first report, showed that Iranian nursing students experience a great deal of vertical violence in the verbal, psychological, and cultural forms. Studies have claimed that any kind of violence or disrespectfulness among students can affect their clinical learning and their professional attitude.^30,31^

It was found that the student's reaction to violence was on a range from counteracting to considering as commonplace. In line with this study, a qualitative study in Iran suggested that students had different reactions to clinical violence which included disregarding, reaction and reporting to an educator.^31^ Although considering violence as commonplace has been well explained among nurses,^10,32^ this study shows that a significant number of nursing students also use this technique against nurses, as a significant strategy. In this regard, the results of a study in Turkey also indicated that students avoided from responding to violence.^19^ A study conducted in Australia and the UK also confirmed the acceptance of bullying in the clinical setting by students.^4^ In addition, the results of this study revealed that nursing students who are the victims of workplace violence do not receive appropriate support from clinical educators and head nurses.

Nursing students reported many psychological aspects as the results of violence. In previous studies, the reported psychological complications such as embarrassment,^33^ waiting for retaliation, hostility,^10,21^ exhaustion,^34^ irritability and post-traumatic stress disorder^6^ caused by workplace violence were more severe than the psychological outcomes reported in this study. More importantly, students did report no physical harm as one of the results of violence and the physical symptoms experienced by the nursing students were due to psychological stress induced by violence.

The findings significantly showed that the experience of violence could have notable educational implications such as a decline in clinical learning and reduced relationship between students and health care personnel. The experience of violence can bear some serious professional implications, including reduced quality of nursing care, increased number of medical mistakes, and downgrading professional attitudes and even promoting a desire to quit the profession. Although there are a few studies in the literature examining the educational and professional outcomes of workplace violence against student, few studies have shown that the experience of workplace violence can lead to a decrease in students’ productivity and the establishment of negative attitude towards nursing and even the desire to quit nursing profession.^6,14,30,35^

## Conclusion

The results of this study suggested that clinical violence experience is a common phenomenon among nursing students which is undertaken by nurses, other professional staff, patients and their caregivers. Violence brings negative psychological and educational consequences for nursing students, which may affect their motivation to stay in the profession. It was also found that the students’ reaction to violence was converted from counteracting and reporting to disregarding and considering as commonplace as they passed several academic semesters and their clinical experience enhances. 

**Acknowledgement**

The authors wish to acknowledge all of the nursing students, whose contribution enabled the production of this article.
